# MMP9: Link between neuropathy and colorectal cancer?

**DOI:** 10.3389/fmolb.2024.1451611

**Published:** 2024-11-27

**Authors:** Cynthia Rosa Regalado, Mihály Balogh

**Affiliations:** Department of Molecular Pharmacology, Groningen Research Institute of Pharmacy, Faculty of Science and Engineering, University of Groningen, Groningen, Netherlands

**Keywords:** MMP9, colon cancer, colorectal cancer, CIPN, matrix metalloproteinase 9, cancer neuroscience, neuropathy

## Abstract

As chemotherapy is still a cornerstone of colorectal cancer (CRC) treatment, chemotherapy-induced peripheral neuropathy (CIPN) presents significant clinical challenges, affecting millions worldwide. A subset of colon cancer patients (approximately 30%) develop chronic CIPN, with detrimental, untreatable neuropathic pain symptoms. The risk factors of such intractable chronic CIPN are unknown. However, there is growing literature data investigating the intriguing interplay of neurons and cancer (cancer neuroscience). Recent data shows that this interplay might have a key role in the development and severity of CIPN. Given its vast (patho)physiological roles in both colon cancer and neuropathy, MMP9 seems to be a key factor that might drive the development of neuronal damage in colon cancer patients. This review investigates the role of matrix metalloproteinase 9 (MMP9) in linking CRC to neuropathy, aiming to uncover shared mechanisms that could offer new therapeutic targets. By synthesizing insights from a broad range of studies published over the last 20 years, we explore MMP9’s involvement in CRC progression, its role in CIPN, and the interconnected pathways influencing both conditions. These studies reveal MMP9 as a pivotal mediator in ECM remodeling, inflammation, and signal transduction pathways, emphasizing its modulation by macrophages. These shared mechanisms of colon cancer and CIPN pathophysiology suggest MMP9’s potential contribution to neuropathic conditions in CRC patients, positioning it as a critical factor in disease progression and a promising therapeutic target. Future research should focus on longitudinal studies to assess MMP9’s impact on neuropathy outcomes in CRC patients, exploring MMP9 inhibitors, and developing targeted interventions to mitigate the detrimental symptoms of CIPN. MMP9 also seems to be a feasible driving factor in the development of chronic CIPN in colon cancer patients.

## 1 Introduction

Colorectal cancer (CRC) poses a significant global health challenge, with over 1.9 million new cases and 930,000 deaths reported in 2020. Projections indicate a dramatic increase by 2040, with an estimated 3.2 million new cases and 1.6 million deaths annually, marking a 63% increase in incidence and a 73% increase in mortality from 2020. This increase is expected mainly in countries with a high Human Development Index, despite effective screening programs in high-income countries. The incidence among younger adults has also risen, leading to the recommended age for average risk screening being lowered from 50 to 45 years in 2020, reflective of shifting risk profiles and the need for early intervention. Contributing factors include a more Westernized diet and increasing rates of overweight and obesity (Morgan et al., 2023). These demographic trends impose a significant financial burden on patients, who face high treatment costs, long-term care expenses, and productivity losses due to illness. Many patients experience financial hardship, with some going into debt or filing for bankruptcy as a result of cancer-related expenses (Orangio, 2018).

Standard screening methods include lower endoscopy and fecal occult blood tests, which, alongside lifestyle modifications, form the cornerstone of risk reduction strategies. Most colorectal tumors evolve from pre-cancerous polyps (adenomas), potentially developing into malignant tumors (carcinomas) over time. Given that the adenoma-carcinoma sequence’s progression to CRC takes about 10 years, there’s a substantial window for early detection and intervention. Through endoscopic procedures or surgery, colorectal adenomas can be removed, effectively preventing cancer formation and offering a potential reduction in the long-term economic impact of CRC (Goel et al., 2022).

Despite the promise of screening and early intervention, CRC remains challenging to diagnose in the early stages. Even after surgery and subsequent treatment, there is a risk of recurrence. Treatment options vary by cancer stage, with stage III, IV, and recurrent CRC typically needing chemotherapy. Treatment regimens range from single-agent therapy (fluoropyrimidine (5-FU) to multiple-agent therapy, including oxaliplatin, irinotecan, and capecitabine, with combination approaches used in first-line treatment to enhance efficacy. However, these treatments come with several drawbacks including systemic toxicity, resistance development, low tumor-specific selectivity, and peripheral nerve damage (Dekker et al., 2019; Goel et al., 2022). The latter manifests as chemotherapy-induced peripheral neuropathy (CIPN), a common and debilitating side effect affecting patients treated with a wide range of antineoplastic agents (Sałat, 2020).

CIPN is characterized by symptoms such as sensory loss, hyperalgesia, allodynia, and paresthesia, resulting from axonal degradation, specifically the loss of large myelinated, small unmyelinated, and intra-epidermal nerve fibers. These adverse effects are often dose-dependent, with oxaliplatin, in particular, known for causing both immediate neurotoxic effects and chronic sensory neuropathy. This axonal damage underlines the motor and sensory impairments experienced by patients (Flatters et al., 2017; Sałat, 2020). The impact of CIPN extends beyond the immediate discomfort; affecting CRC survivors long after treatment. A recent systematic review reported that approximately 58% of patients experience CIPN at 6 months post-chemotherapy, with the prevalence gradually decreasing to 24% at 3 years, yet a significant portion still endures long-term symptoms (Teng et al., 2022). In a study of 86 CRC patients, 37.2% reported CIPN during chemotherapy, which only slightly decreased to 32.6% 3 months after treatment, with ongoing disturbances in daily activities such as sexual life and exercise, and contributing to depression in 23.5% of patients (Oh et al., 2020). This persistence of symptoms, known as coasting, where neuropathy worsens or remains unchanged after chemotherapy end, significantly affects survivors’ quality of life and underscores the need for effective management strategies in post-treatment care (Flatters et al., 2017). The specific factors that might drive the development of these late symptoms are unknown.

The American Society of Clinical Oncology guidelines currently do not recommend any agents for the prevention of CIPN due to a lack of effective evidence. However, duloxetine has been moderately recommended for treating painful CIPN, based on trial evidence. This specific recommendation highlights the challenge of managing CIPN, as most conventional systemic treatments, such as antiepileptic agents, antidepressants, and opioids, lack conclusive clinical efficacy for CIPN (Maihöfner et al., 2021). The variability in symptom severity among patients further complicates CIPN management, emphasizing the need for personalized treatment plans. These plans might include dose adjustments, symptomatic relief measures, and physical therapy to maintain muscle strength and function (Flatters et al., 2017).

The pathophysiology of CIPN is multifaceted, involving cellular oxidative stress, dysregulated calcium homeostasis, neuroinflammation, axonal degradation, and immune system involvement. This complexity highlights the need for extensive research to identify potential therapeutic targets (Sałat, 2020). Among these, matrix metalloproteinase-9 (MMP9) has attracted considerable attention for its potential role in both CRC progression and CIPN.

Emerging research indicates that the development of colon tumors may inherently disrupt systemic neuronal function in a manner akin to CIPN, even in the absence of chemotherapeutic agents. Such dysfunction has been linked to increased levels of inflammatory mediators, including chemokines like CCL2, CXCL1, CXCL2, and notably CXCL10, which may be important in the onset of chronic CIPN developing in some colon cancer patients. In addition, systemic MMP9 levels were also shown to be increased in colon tumor bearing mice (Balogh et al., 2022). These colon cancer bearing mice were showing various symptoms of neuropathy, without any chemotherapeutic treatment. These findings suggest that colon cancer may initiate complex inflammatory responses, leading to neuronal changes. Within this complex interplay, matrix metalloproteinases (MMPs) emerge as significant players. As a family of zinc-dependent endopeptidases, MMPs are implicated in processes such as cancer metastasis, chronic inflammation, and neurological disorders. They are secreted by various types of connective tissue and pro-inflammatory cells, playing a crucial role in the degradation of extracellular matrix components, facilitating not only tumor growth and metastasis but potentially also contributing to the pathophysiology of CIPN (Pezeshkian et al., 2021).

Among the MMP family, MMP9, or gelatinase B, has garnered attention for its overexpression and upregulation in various cancerous conditions. Its enzymatic activity on type IV collagen, a fundamental element of the basement membrane, is pivotal for cell transformation and carcinogenesis (Mondal et al., 2020). In the context of colorectal cancer, MMP9 has been associated with tumor growth and metastasis (Mudatsir et al., 2023), while in neuropathic conditions like CIPN, it is implicated in neuronal damage (Dai et al., 2024). However, the exact mechanisms by which MMP9 exerts these effects in both cancer and neuropathy are yet to be fully elucidated, highlighting the need for further study.

Tumor cells, through a paracrine mechanism involving the secretion of interleukins, interferons, growth factors, and other MMP inducers, can prompt neighboring host cells to produce MMPs necessary for cancer progression. MMPs, secreted by the non-malignant cells of the surrounding tissue, can be appropriated by cancer cells, binding to their surfaces and serving the tumor’s invasive requirements. MMPs are generally produced as inactive proenzymes (pro-MMPs) and are activated through cleavage processes. For MMP9, this involves two specific cleavage steps to become active. This activation is tightly regulated by tissue inhibitors of metalloproteinases (TIMPs) and involves various activators, with MMP3 being notably potent. Therefore, the regulation of MMP9 centers on a delicate balance between the activation of its proenzyme form and the counteraction of TIMPs, underscoring the complexity of its regulatory dynamics and the interplay with the extracellular matrix (ECM) (Mondal et al., 2020).

A deeper understanding of MMP9’s role in the pathogenesis of colorectal cancer and its associated neuropathic conditions is essential to anticipate patient outcomes and develop novel therapeutic approaches. While individual studies have addressed CRC pathogenesis and CIPN mechanisms, a direct exploration of MMP9 as a shared factor between CRC and CIPN remains uncharted, underscoring a significant gap in the literature. This review aims to assess the role of MMP9 in the progression of colorectal cancer (CRC) and the development of chemotherapy-induced peripheral neuropathy (CIPN). It explores the shared mechanisms involving MMP9, focusing on the interplay between inflammatory mediators, signaling pathways, and MMP9. Additionally, the review explores whether MMP9 upregulation in CRC might be a risk factor for the development of CIPN. The insights from this review aim to expand our understanding and direct future research into the complex relationship between MMP9, CIPN, and colorectal cancer.

## 2 MMP9 and the pathophysiology of colorectal cancer

### 2.1 Changes in MMP9 levels and the development of colorectal cancer

MMP9 plays a crucial role in the progression of CRC. The diagnostic potential of serum MMP9 levels has been highlighted by meta-analysis data, which found that individuals with CRC exhibit significantly higher levels of MMP9 in their serum compared to healthy controls, with sensitivity and specificity around 69% and 68%, respectively. This positions MMP9 as a critical molecular player in CRC detection and monitoring (Liang and Chang, 2018).

A cross-sectional study focusing on the relationship between MMP9 expression and clinicopathological features in CRC patients reinforces MMP9 as a biomarker. Through the analysis of tissue samples from 52 patients, the study reveals that higher MMP9 expression is linked to more advanced cancer stages and higher grades of tumor pathology. It was observed that the stroma surrounding the tumor overexpresses MMP9, which might be a response to factors released by tumor cells or other components of the tumor microenvironment. This overexpression contributes to the remodeling of the tissue architecture, favoring tumor invasion and possibly angiogenesis, essential processes for metastasis. Supporting this, evidence from *in-vitro* studies demonstrates how colorectal cancer cell lines can trigger MMP9 production in monocytes through a membrane barrier, illustrating the non-direct communication within the tumor microenvironment. Local basement membrane deterioration, a prerequisite for endothelial and tumor cell infiltration, is a function in which MMPs, including MMP9, are actively involved. Furthermore, the absence of MMP9 compromises the invasive capabilities of tumors, solidifying MMP9’s position as not only a marker for CRC aggressiveness but also as a promising target for therapeutic intervention (Mudatsir et al., 2023).

Observational data from 31 cases of stage III colorectal adenocarcinomas reveal significant but inconsistent MMP9 expression across different tumor cells, with higher levels in poorly and moderately differentiated carcinomas compared to well-differentiated ones. High MMP9 activity in metastasis-free lymph nodes suggests its involvement in preparing these sites for future metastatic spread and a potential role in immune modulation (Florin Georgescu et al., 2015).

MMP9 is also tied to angiogenesis intensity and may contribute to the angiogenic switch, the transition from a non-angiogenic to an angiogenic phenotype in tumors. Tumor cells, along with peritumoral macrophages and stromal cells, contribute to the dynamic changes in the ECM by expressing MMP9. By degrading type IV collagen and denatured collagen molecules, MMP9 facilitates tumor cell invasion into surrounding tissues and metastasis. Alterations in the ECM mediated by MMP9 lead to substantial architectural modifications within the tumor stroma, resulting in a desmoplastic reaction. This desmoplastic stroma is common in more than 50% of colorectal cancers, suggesting that MMP9’s activity in stromal remodeling is associated with a more aggressive tumor phenotype (Florin Georgescu et al., 2015).

The prognostic value of MMP9 is further emphasized by clinical studies showing substantial upregulation of MMP9 in cancerous tissues compared to normal mucosa. Elevated MMP9 levels correlate strongly with lymph node metastasis and higher Dukes' stages, indicating its significant role in tumor invasion and metastasis. Survival analysis indicates that patients with higher MMP9 expression have significantly lower survival rates, reinforcing its importance as an independent prognostic marker (Yang et al., 2014).

Advancements in MMP9-targeted therapy underscore its therapeutic potential. The development and preclinical evaluation of GS-5745, a highly selective allosteric inhibitor in the form of a humanized monoclonal antibody, have demonstrated efficacy in reducing tumor growth and metastasis without the common toxicity associated with less selective MMP inhibitors. In preclinical models, GS-5745 effectively decreased disease severity in ulcerative colitis and reduced primary tumor growth and metastasis incidence in colorectal cancer. These findings suggest that MMP9 plays a critical role in the pathways involved in growth, inflammation, and cancer metastasis by acting not only as a downstream effector but also as an upstream mediator of these processes. The selective inhibition of MMP9 thus presents a promising therapeutic avenue for impacting CRC progression (Marshall et al., 2015).

Increased levels of IL-6 and C-reactive protein (CRP) alongside MMP9 with advancing CRC stages indicate the role of systemic inflammation in modulating the tumor microenvironment and driving cancer progression. IL-6’s signaling mechanisms are particularly relevant, as this cytokine can engage in both classic signaling and trans-signaling pathways. In trans-signaling, IL-6 binds to a soluble form of its receptor and subsequently engages the gp130 receptor subunit on cell surfaces, which activates the JAK/STAT pathway. This process promotes tumor cell proliferation and inhibits apoptosis. The significant correlation between CRP and MMP9 levels further indicates that systemic inflammation, as reflected by CRP, is closely associated with MMP9 activity (Rasic et al., 2018).

Collectively, these findings provide a comprehensive view of MMP9’s involvement in CRC. Elevated MMP9 levels are consistently linked to more aggressive tumor behavior, advanced disease stages, and poorer patient outcomes. The enzyme’s role in ECM degradation, stromal remodeling, and immune modulation contributes multifaceted to cancer progression. These insights not only establish MMP9 as a critical biomarker and prognostic factor but also highlight its potential as a therapeutic target, paving the way for novel clinical interventions in CRC.

#### 2.1.1 Chemotherapy-induced modulation of MMP9 in CRC

Studies using a 3D tumoroid model of metastatic CRC demonstrate that cisplatin and 5-FU, commonly used chemotherapeutics, stimulate MMP9 promoter activity within residual cancer cells, despite differing effects on overall tumor growth. Specifically, 5-FU effectively inhibited tumor growth by reducing the proliferation of tumor cells within the model, whereas cisplatin was less effective and, in some instances, even promoted tumor growth. This unexpected promotion of growth by cisplatin may result from mechanisms involving exosome-mediated drug expulsion. Nonetheless, both drugs increased MMP9 activity, potentially contributing to an invasive phenotype among resistant cells. Notably, 5-FU treatment, while reducing tumor bulk, left behind MMP9-positive dormant cells, which may later drive invasion and metastasis (Sogawa et al., 2021). In addition to single agent chemotherapies, combination treatments can variably affect MMP9 expression. A study using 17-AAG (also named as Tanespimycin) with capecitabine (Cap) or irinotecan (IR) demonstrated a marked downregulation of MMP9 and VEGF in CRC cells, particularly in HT-29 cells. Cap/17-AAG combination was especially effective, suggesting a strong anti-metastatic and anti-angiogenic effect when used together. For IR/17-AAG, MMP-9 and VEGF downregulation was observed but to a lesser extent. Although a triple combination (Cap, IR, and 17-AAG) was tested, it was less effective in downregulating MMP-9 and VEGF and did not significantly inhibit cell migration, suggesting that the double combinations are more effective for anti-metastatic and anti-angiogenic effects in CRC (Zeynali-Moghaddam et al., 2019). These findings highlight the variable impact of different chemotherapy agents on MMP9 levels, suggesting that while some single-agent treatments may inadvertently upregulate MMP9 and promote invasion through residual, dormant cells, combination therapies that downregulate MMP9, like Cap/17-AAG, may offer enhanced anti-metastatic benefits.

The variability in MMP9 expression across different chemotherapy treatments suggests that its levels are not consistent across all contexts and may change depending on the specific drugs or combinations used. This presents a challenge for using MMP9 as a consistent predictive marker, as its expression might not consistently reflect tumor behavior across various treatment regimens. In clinical practice, these fluctuations could limit MMP9’s effectiveness as a universal marker for monitoring tumor response or predicting treatment resistance. However, MMP9 may be more effective as a marker in specific chemotherapy contexts, where its expression changes are more predictable. Further research is needed to identify these specific contexts and determine how MMP9 could best be used as a clinical marker, especially in tailored treatments where controlling MMP9 expression aligns with therapeutic goals.

### 2.2 Signaling pathways regulating MMP9 in colorectal cancer

**FIGURE 1:**
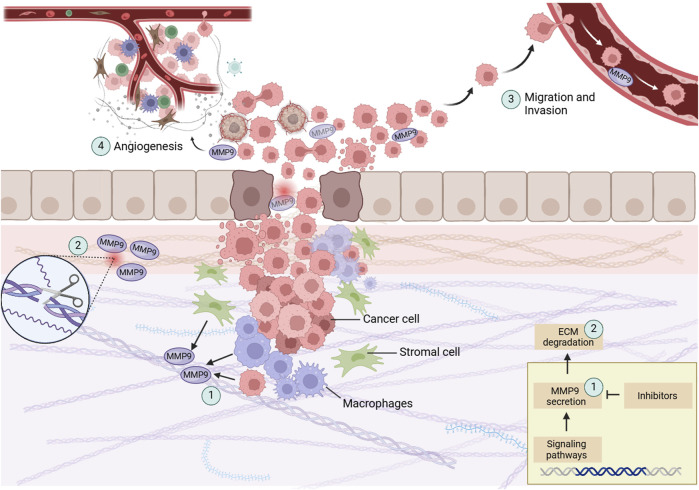
This illustration showcases the central processes in colon cancer progression with a focus on MMP9. (1) MMP9, secreted by cancer cells, stromal cells, and peritumoral macrophages, play a crucial role in tumor dynamics. (2) By degrading the ECM, represented here as fragmented collagen fibers, MMP9 aids in the breakdown of structural barriers, allowing tumor cells to invade adjacent tissues. (3) The facilitation of tumor cell migration and invasion, depicted through the disrupted basement membrane, further demonstrates MMP9’s role in metastasis. (4) MMP9 is involved in the angiogenic switch during carcinogenesis, illustrated by new blood vessels forming towards the tumor, showing its contribution to tumor growth and nutrient supply. Created with BioRender.

Multiple papers clarify MMP9’s pivotal role in the signaling pathways that drive CRC progression. The expression of MMP genes is intricately regulated by various stimulatory factors, including cytokines and tumor promotors, which exert their influence through signal transduction pathways. The intricate interplay of these pathways highlights the regulatory environment controlling MMP9 expression. The multifaceted roles of MMP9 in colon cancer growth are illustrated in Figure 1.

It has been demonstrated that the JAK2/STAT3 signaling pathway is involved in the regulation of MMP9 expression in colorectal cancer. Activation of this pathway by B7-H3, a co-stimulatory molecule known for its elevated expression in various cancers, significantly upregulates MMP9. This upregulation promotes cell migration and invasion, contributing to CRC progression. Inhibition of this pathway with AG490, a selective JAK2 inhibitor, results in decreased MMP9 expression. Therefore, MMP9 is a downstream effector of the B7-H3/JAK2/STAT3 axis and targeting this axis might mitigate CRC invasion and metastasis (Liu et al., 2015).

The Wnt/β-catenin pathway also plays a role in regulating MMP9. Overexpression of USP39, a spliceosome-associated protein, enhances this pathway and leads to increased cancer cell growth, migration, and invasion. Suppressing USP39 reduces cell proliferation and invasion capabilities by downregulating key components of the Wnt/β-catenin pathway, including β-catenin, TCF4, MMP2, and MMP9. Thus, MMP9 operates as a downstream target in the USP39-mediated activation of the Wnt/β-catenin signaling pathway (Yuan et al., 2017). Studies have shown that treatments targeting this pathway can effectively reduce MMP9 levels. For instance, the administration of cinobufacini notably decreases MMP2 and MMP9 levels, thereby inhibiting cancer cell migration and invasion. Therefore, modulating the Wnt/β-catenin pathway could be a therapeutic strategy to control MMP9 expression and combat CRC progression (Wang et al., 2020).

Furthermore, the extracellular signal-regulated kinase (ERK) pathway plays a significant role in CRC progression through its regulation of MMP9 expression. Studies have shown that modulating this pathway can impact cancer cell behavior. For instance, research demonstrates that sevoflurane, an inhalational anesthetic, reduces CRC cell migration and invasion by downregulating ERK phosphorylation, leading to decreased MMP9 expression. This effect is further enhanced by the upregulation of miR-203, which targets the ERK pathway, putting MMP9 as a downstream effector in this signaling cascade (L. Fan et al., 2019). In a complementary study, it was found that β6 integrin upregulation enhances MMP9 expression via the ERK pathway. This process involves the formation of a signaling complex with ETS1, a transcription factor that binds to the MMP9 promoter, boosting its transcription. The inhibition of ERK phosphorylation or ETS1 knockdown effectively reduces MMP9 levels (Gao et al., 2014). Additionally, another study on the novel derivative EnDuo shows its inhibitory effects on MMP9 expression by disrupting both the PI3K/AKT and ERK pathways. This underscores the interconnected nature of these signaling pathways in regulating MMP9 and their importance in CRC progression (Idiiatullina et al., 2021). Collectively, these findings emphasize the unidirectional influence of the ERK pathway on MMP9 as a downstream target, providing insights into potential therapeutic strategies for mitigating CRC invasion and metastasis.

Research has demonstrated the potential of targeting the PI3K/AKT to mitigate CRC progression. The study of KiSS-1, a metastasis-suppressing gene, illustrates how its overexpression can significantly reduce MMP9 expression, thereby inhibiting cell invasion. This mechanism operates through the PI3K/AKT/NF-κB pathway, with KiSS-1 not only hampering cell proliferation but also enhancing apoptosis. The application of PI3K and AKT agonists to counteract KiSS-1’s effects further confirmed the pathway’s central role in these processes, positioning KiSS-1 as a promising target for CRC treatment (Chen et al., 2016).

In contrast, Benzyl Isothiocyanate (BITC) demonstrates the inhibition of multiple signaling pathways, including PKCδ, JNK1/2, ERK1/2, and PI3K, which are crucial for NF-κB activation and subsequent MMP9 synthesis. BITC’s ability to suppress these pathways leads to reduced NF-κB DNA binding activity, directly downregulating MMP9 transcription. This reduction in MMP9 expression is achieved through the inhibition of PI3K and AKT, thereby decreasing the invasive potential of colon cancer cells. Upon PKC activation, there is a subsequent activation of the MAPK signaling pathways, specifically JNK1/2, P38, and ERK1/2, as well as the PI3K pathway. These pathways modulate transcription factors such as AP-1, NF-κB, and Sp-1, which regulate the expression of MMP9. By inhibiting these pathways, BITC effectively reduces the invasive potential of HT29 colon cancer cells (Lai et al., 2010).

Conversely, cholic acid (CA) activates the NADPH oxidase system in colon cancer cells, leading to increased reactive oxygen species (ROS) production and the activation of MAPK signaling pathways. Unlike BITC’s inhibitory action, CA enhances MMP9 expression through the upregulation of transcription factors AP-1 and NF-κB. These factors bind to their respective sites on the MMP9 gene, promoting cellular invasion. The study’s findings that inhibitors of ERK1/2, JNK, and p38 MAPK can partially block CA-induced MMP9 expression underscore the crucial role of these kinases in mediating MMP9’s involvement in cancer cell invasion (Li et al., 2020).

Studies have demonstrated the diverse effects of different p38 MAPK isoforms on MMP9 expression. For instance, research on Taiwanin E, a phytoestrogen compound, reveals that it inhibits cell migration by modulating MMP9 expression through the activation of the p38 MAPK pathway. Taiwanin E treatment increases the phosphorylation of p38, particularly the p38α isoform, leading to a dose-dependent suppression of MMP2 and MMP9 expression and activity. Inhibition or knockdown of p38α significantly reduces the compound’s inhibitory effects on cell migration, underscoring the crucial role of p38α in this process (Hsu et al., 2017). In contrast, the p38γ isoform has been identified as a promoter of oncogenesis. Research shows that p38γ enhances c-Jun synthesis and MMP9 transcription, thereby promoting cell invasion. This isoform requires phosphorylation and its C-terminus to bind c-Jun, which is necessary for MMP9 trans-activation. The activated p38γ/c-Jun/MMP9 complex observed in human colon cancer tissues links these laboratory findings with clinical observations, illustrating that p38γ acts as both an activator and a cofactor of a transcription factor, which diversifies potential therapeutic approaches in CRC. And so, the p38 MAPK pathways can have differing and sometimes opposing effects on tumor biology depending on the isoform involved (Loesch et al., 2010).

Additionally, protease-activated receptor 2 (PAR2) activation influences MMP9 regulation in CRC. Activation of PAR2 by its agonist or coagulant Factor VIIa leads to a notable increase in MMP9 and CD44 expressions while reducing the expression of caspase-3, a pro-apoptotic enzyme. This suggests a mechanism by which cancer cells might resist apoptosis. This study by Wu et al. links thrombosis and cancer progression through tissue factor (TF), promoting cancer progression by activating the local coagulation cascade and engaging cell-signaling pathways. This PAR2-centric pathway offers a new perspective on the multifaceted regulation of MMP9, proposing that the local coagulation environment within the tumor contributes to the upregulation of MMP9, thereby enhancing the invasive capabilities of CRC cells (Wu et al., 2013). The aforementioned preclinical studies were all conducted *in vitro* using CRC cell lines.

Based on the preceding discussion, the role of MMP9 in colorectal cancer pathogenesis is central to understanding the disease’s aggressive nature. MMP9 is regulated by several key signaling pathways, including JAK2/STAT3, Wnt/β-catenin, ERK, and PI3K/AKT, each contributing to its upregulation and the progression of cancer (Fan et al., 2019; Liu et al., 2015; Yuan et al., 2017). For instance, the ERK pathway modulates MMP9 expression, with agents like sevoflurane reducing ERK phosphorylation and subsequently MMP9 levels (Fan et al., 2019). MMP9’s interaction with β6 integrin via the ERK/MAPK pathway underlines the importance of cell adhesion and extracellular matrix remodeling in CRC metastasis (Gao et al., 2014).

The MAPK family, including ERK, JNK, and particularly p38 MAPK, plays a comprehensive role in adjusting MMP9 levels in response to various cellular signals. Activation of these pathways upregulates transcription factors such as AP-1, NF-κB, and Sp-1, which directly influence MMP9 expression (Lai et al., 2010). The p38 pathway has a unique role in modulating MMP9 expression, differing from the tumor-suppressive effects attributed to its other isoforms (Hsu et al., 2017). The PI3K/Akt pathway is another significant regulatory avenue, where its blockade or inhibition correlates with decreased MMP9 levels (Chen et al., 2016; Lai et al., 2010). Moreover, MMP9’s responsiveness to oxidative stress through ROS interaction and its regulation via PAR2 activation add layers to its role in modulating the tumor microenvironment and promoting invasiveness (Li et al., 2020; Wu et al., 2013).

These pathways elucidate MMP9’s modulation both directly and indirectly by various stimuli, acting as a key downstream effector in signaling cascades. This integrative role of MMP9 connects upstream signals to facilitate tumor invasion, metastasis, and angiogenesis, pointing to its potential as a target for therapeutic interventions aimed at disrupting its pro-tumorigenic functions (see Figure 1).

### 2.3 Summarizing the roles of MMP9 in colon cancer development

It is evident that MMP9 directly influences CRC progression in several ways. Elevated serum levels of MMP9 serve as a potential diagnostic marker, reflecting the tumor’s burden and aggressiveness (Liang and Chang, 2018). Overexpression in tumor tissues and the surrounding stroma is a hallmark of advanced disease, actively facilitating the tumor’s invasive capacity and subsequent metastasis (Mudatsir et al., 2023). Particularly in poorly differentiated carcinomas, MMP9’s expression pattern implicates it in the remodeling of the extracellular matrix and angiogenesis, which are crucial processes for tumor aggressiveness (Florin Georgescu et al., 2015). The clinical implications of MMP9 upregulation, correlating with poorer patient outcomes, further emphasize its significance as a prognostic factor and a potential lever in therapeutic decision-making (Yang et al., 2014).

The development and efficacy of selective MMP9 inhibitors in preclinical studies highlight the enzyme’s viability as a therapeutic target, offering a promising strategy to attenuate CRC progression (Marshall et al., 2015). MMP9’s association with inflammatory mediators throughout CRC stages hints at its broader implication in tumor-related inflammation and tissue alterations (Rasic et al., 2018). Collectively, these insights into MMP9’s regulatory mechanisms and its consequential role in CRC underline the enzyme’s critical involvement in the disease’s pathogenesis. Understanding MMP9’s function in CRC not only sheds light on the disease’s underlying molecular dynamics but also opens avenues for targeted therapeutic interventions, aiming to disrupt its pro-tumorigenic activities and improve patient outcomes.

## 3 MMP9’s involvement in chemotherapy-induced peripheral neuropathy

### 3.1 MMP9 and neuronal damage: Results of various neuropathic pain studies

**FIGURE 2:**
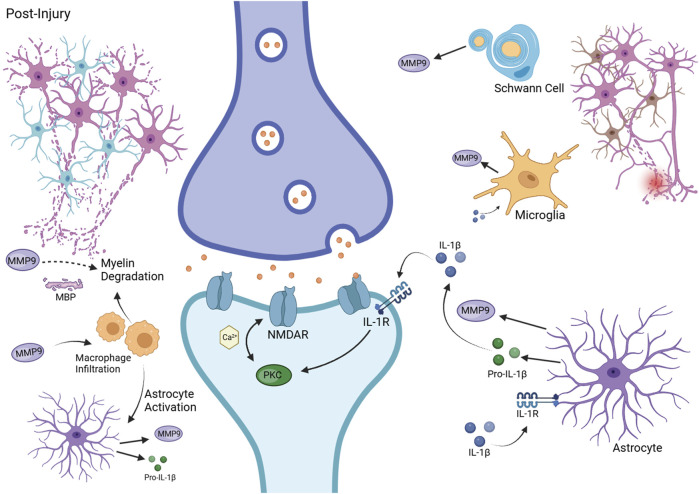
MMP9 in Neuropathic Pain Pathophysiology. This figure depicts the contribution of MMP9 to neuropathic pain following injury. It shows MMP9 release from Schwann cells, microglia, and astrocytes, its role in pro-IL-1β activation, and the ensuing neuronal sensitization and myelin degradation. Additionally, it illustrates macrophage infiltration and astrocyte activation, highlighting the inflammatory and neuropathic responses. Created with BioRender.com.

Exploring MMP9’s role in neuropathic pain and neuronal damage involves understanding its specific mechanisms, across various neuropathic conditions, not just chemotherapy-induced peripheral neuropathy (CIPN). This approach allows for the potential translation of findings from varied neuropathic conditions to the context of CIPN and colon cancer. Figure 2 illustrates the various mechanisms by which MMP9 might drive systemic neuronal damage.

Preclinical studies on the effects of N-acetyl-cysteine (NAC) and procyanidins (PC) reveal MMP9’s involvement in neuropathic pain through the modulation of pro-inflammatory cytokines, such as IL-1β, which are crucial for the development of central sensitization by enhancing the hyperactivity of dorsal horn neurons. The role of microglia-released cytokines, including IL-1β, TNF-α, and IL-6, is particularly significant, with IL-1β playing a pivotal role in inflammatory processes (Li et al., 2016; Pan et al., 2018).

Using chronic constrictive injury (CCI) model, studies show that nerve injury prompts the release of MMP9 and pro-IL-1β into the extracellular matrix. MMP9 facilitates the conversion of pro-IL-1β into its active form, inducing hyperexcitability in nociceptive neurons. NAC and PC influence MMP9 expression, potentially through the inhibition of pathways like JNK and p38 MAPK. Additionally, ROS produced during inflammation and injury can activate MMP9. Inhibition of MMP9 blocks the maturation of IL-1β, thereby reducing hyperexcitability in dorsal root ganglion (DRG) neuronal cell bodies and axons, leading to decreased peripheral sensitization. The ROS scavenging abilities of NAC and PC might also contribute to the inhibition of MMP9 expression via the modulation of the AP-1 and NF-κB pathways, which are implicated in the pro-inflammatory state associated with neuropathic pain. Both studies emphasize the ‘cysteine switch' mechanism, which prevents the oxidation of the critical cysteine residue on MMP9 (Li et al., 2016; Pan et al., 2018).

Notably, NAC prevents the phosphorylation of key MAPK family proteins and reduces microglial activation, while PC significantly reduces the phosphorylation of neuron activation markers PKCγ and NR1. PC also diminishes MMP9/2 activity and suppresses LPS-induced p38 and NF-κB signaling in microglia, indicating that PC could modulate MMP9/2 function at both the protein and transcriptional levels (Pan et al., 2018).

The naturally occurring flavonoid isoorientin suppresses MMP9 activation by reducing the levels of key pro-inflammatory cytokines, including IL-6, IL-1β, and TNF-α. This suggests that inhibiting MMP9 not only impacts downstream inflammatory responses but also supports the potential therapeutic value of these compounds in managing neuropathic pain (Zhang et al., 2019).

Further exploration into MMP9’s mechanisms reveals the activation the JNK-MMP9 pathway in astrocytes following CCI of the sciatic nerve. MMP9-mediated conversion of pro-IL-1β to its active form facilitates PKCγ phosphorylation through IL-1β receptors, enhancing NMDA receptor activity and contributing to central sensitization. This process also increases proinflammatory cytokine production, sustaining neuropathic pain. Tetramethylpyrazine suppresses this pathway by inhibiting upstream regulators of JNK, such as TAK1, thereby reducing MMP9 expression in the spinal cord. The differential activation of MAPK family members in response to nerve injury, with ERK activation predominantly in neurons, p38 in microglia, and JNK in astrocytes, highlights the intricate nature of neuropathic pain and the importance of targeted molecular approaches for effective pain management (Jiang et al., 2017).

Peripheral nerve injury studies have shown that MMP9 expression in Schwann cells significantly increases, leading to axonal degeneration and macrophage recruitment to the lesion site. Research using rat and mice models of sciatic nerve crush injury demonstrates that pro-inflammatory cytokines such as TNF-α and IL-1β activate glia, prompting MMP9 production in the DRG after injury. MMP9 plays a role in regulating myelin basic protein (MBP) turnover and myelin thickness, with MMP9 gene deletion protecting against neuropathic pain and preventing myelin degradation (Chattopadhyay et al., 2007).

These findings are supported by studies showing MMP9’s involvement in MBP degradation within Schwann cells, preceding mechanical allodynia. Kobayashi and colleagues explain that when a peripheral nerve is injured, pro-inflammatory cytokines serve as signals that alert Schwann cells to the presence of nerve injury. In response, Schwann cells upregulate the production of MMP9, which then plays a critical role in the nerve injury response, including myelin degradation and the propagation of inflammation, which can contribute to neuropathic pain. The degradation of MBP occurs quickly, within a day of the injury, leading to Schwann cell-mediated demyelination. The degradation of the myelin sheath exposes the axonal plasma membrane of the myelinated Aβ fibers. Normally, myelin protects the nerve fibers from inappropriate stimuli. However, when the myelin is compromised, the axonal membrane becomes susceptible to ectopic hyperexcitability and responds abnormally to mechanical stimuli that are not typically painful. Additionally, MMPs promote the infiltration of macrophages which contribute to further axonal demyelination and degeneration and are also implicated in the activation of astrocytes, which are central for the maintenance of peripheral neuropathic pain. The study showed that in MMP9 knockout mice the MBP was preserved after injury, indicating the crucial role of the enzyme in the degradation process. Furthermore, treatment with GM6001, a broad-spectrum MMP inhibitor, prevented MBP degradation, reduced the infiltration of macrophages at the injury site, inhibited astrocyte activation in the spinal cord, improved cell survival, and resulted in immediate and sustained attenuation of mechanical allodynia (Kobayashi et al., 2008).

While MMP9’s impact on myelin integrity has been established, its role in modulating chemokine activity presents another layer of complexity to neuropathic pain mechanisms. Research shows that MMP9 regulates the expression of CX3CL1, which plays a role in microglial activation and pain signal transduction within the spinal cord. Following chronic sciatic nerve constriction injury, both MMP9 and CX3CL1 protein levels rise significantly, suggesting a regulatory relationship. MMP9 may facilitate the transition of CX3CL1 from an inactive, membrane-bound state to an active, soluble form, engaging the CX3CR1 receptor and propagating pain signaling (Zhao et al., 2020).

Building on the concept of MMP9’s regulatory role on CX3CL1, it is broader implication in glial activation becomes clear. Microglia are critical in the onset of neuropathic pain, while astrocytes maintain chronic pain. MMP9 initiates the generation of chemokines and cytokines and activates receptors like CCR2 and CX2CR2, triggering signaling cascades through MAPKs and NF-κB pathways. This culminates in the synthesis of pro-inflammatory mediators, contributing to chronic pain. Paeoniflorin, shown to suppress MMP9 activity in the spinal cord of mice following plantar incision, reduces microglial activation and pro-inflammatory cytokine production, thereby attenuating mechanical allodynia. This effect is mediated by the inhibition of p38 MAPK phosphorylation and modulation of NMDA receptors, suggesting paeoniflorin’s potential in targeting microglial pathways for postoperative pain relief (Fan et al., 2018).

Further, studies on adult CD-1 mice models show temporal MMP9 upregulation in the trigeminal ganglion following CCI, correlating with early pain-related behaviors. Administration of resveratrol prior to CCI curtails MMP9 and MMP2 activity, reducing mechanical allodynia. MMP9 activity is linked to the TLR-4/NF-κB signaling pathway, with resveratrol enhancing SOCS3 expression, mitigating TLR-4/NF-κB mediated signaling, and tempering MMP9/2 expression. This negative feedback mechanism disrupts the inflammatory response and neuropathic pain sensitization (Yin et al., 2019).

Conversely, a study on sciatic nerve CCI in rats shows MMP9 upregulation in Schwann cells 28 days post-CCI, with a decrease in TIMP1 levels. This imbalance towards proteolytic activity is noted at day 1 post-CCI, where high levels of inactive MMP9 proenzyme coincide with elevated TIMP1. However, in the presence of an excess of latent MMP9 over TIMP1, free MMP9 proenzyme not inhibited by TIMP1 is activated by other molecules like plasmin or MMP3, suggesting a prolonged and unregulated proteolytic activity that could contribute to the maintenance and exacerbation of neuropathic pain mechanisms well beyond the acute phase of injury (Remacle et al., 2018).

The collective insights from these preclinical studies, spanning various models and interventions, reinforce MMP9’s central role in the pathophysiology of neuronal damage and neuropathic pain and highlight the diverse mechanisms open to therapeutic intervention (Figure 2). MMP9 modulates the post-injury extracellular environment, promoting myelin basic protein and demyelination, sensitizing axonal membranes and leading to neuropathic pain. It is also important in activating immune cell and orchestrating the inflammatory response, as evidenced by its role in macrophage infiltration and cytokine activation (Chattopadhyay et al., 2007; Jiang et al., 2017; Kobayashi et al., 2008). Particularly, MMP9’s role in cleaving CX3CL1 exemplifies its key position in the modulation of both cytokines and chemokines associated with neuropathic inflammation (Zhao et al., 2020). The enzyme’s interactions with nervous system components, including microglia and astrocytes, further depict its comprehensive influence across both the initiation and maintenance of neuropathic pain states (Jiang et al., 2017; Kobayashi et al., 2008). The suppression of elevated MMP9 activity has been shown to reduce microglial activation and, in turn, the production of pro-inflammatory cytokines, underscoring its potential as a therapeutic target (Pan et al., 2018).

### 3.2 MMP9 and chemotherapy induced neuropathic pain

Turning our focus to CIPN and its molecular mechanism, various studies highlight MMP9’s role in this condition. Research identifies a transcriptional increase in MMP9 alongside a decrease in its inhibitor TIMP1 in the DRG of paclitaxel-treated mice, leading to an imbalance that drives neuroinflammation. Intrathecal injections of a monoclonal antibody against MMP9 (MMP9, mAb, clone 6-6B) prevented and reversed paclitaxel-induced mechanical allodynia but did not mitigate cold allodynia. Interestingly, while MMP9’s role in activating immune cells is known, treatment with MMP9 mAb did not alter activation markers for satellite glial cells and macrophages after 14 days of paclitaxel administration. This suggests that MMP9 inhibition’s protective effects against CIPN may not directly involve modulation of these cell types' activation states. Instead, the therapeutic benefits of MMP9 inhibition seem to manifest through other pathways, notably by reducing proinflammatory cytokines like IL-6 and TNFα, decreasing inducible nitric oxide synthase levels, reducing ROS production, and preserving intraepidermal nerve fibers (Tonello et al., 2019). Additionally, MMP9 amplifies TLR4 signaling by increasing intracellular matrix components, such as biglycans and hyaluronan, which allow tissues to detect non-infectious injuries and translate them into local inflammation. Trimetazidine downregulates TLR4 and MMP9 expression, reducing IL-1β and boosting anti-inflammatory IL-10, indicating its potential to modulate both the onset and progression of CIPN (Hammad et al., 2023).

Chemotherapy not only triggers the release of inflammatory factors but also prompts macrophages to express TF, leading to thrombosis and potentially causing microthrombosis around peripheral nerves. This can induce hypoxia, activating hypoxia-inducible factors (HIFs) and increasing the expression of downstream cytokines like MMPs. In a study on mice with oxaliplatin-induced CIPN, oxaliplatin treatment upregulated TF and activated MMP9/2, contributing to nerve damage and neuropathic pain through thrombosis-mediated circulatory disturbances. This process is exacerbated by the release of Heat Shock Protein 70 (HSP70) and activation of HIF-1α, enhancing MMP expression. The anticoagulant Hirudin countered these effects by inhibiting the overexpression of p-p38 and HIF-1α, and thus MMP9/2 activation. Targeting the HSP70-TLR-4-p38-TF-HIF-1α axis could offer new ways to combat CIPN, although the straightforward application of anticoagulants for CIPN treatment poses risks, particularly due to the potential for severe cardiovascular events in chemotherapy patients with thrombocytopenia (Yang et al., 2019).

Neuroinflammation is a primary mechanism underlying CIPN, with high-mobility group box 1 (HMGB1), categorized as a damage-associated molecular pattern, playing a critical role. Studies have shown that HMGB1 levels and MMP9 activity increase in the plasma of CIPN patients and mice following oxaliplatin treatment. Oxaliplatin triggers HMGB1 release from neurons and stress cells, functioning as an extracellular inflammatory cytokine. Binding to receptors like RAGE and TLRs, extracellular HMGB1 initiates signaling cascades through the NF-κB pathway, enhancing MMP9 expression and exacerbating CIPN (Yang et al., 2023).

Activation of AMP-activated protein kinase (AMPK) facilitates the clearance of HMGB1, reducing MMP9 activity. AMPK activation increases HMGB1 phagocytosis by macrophages via the macrophage scavenger receptor A1 (SR-A1), routing the engulfed HMGB1 to lysosomal degradation. The AMPK/SR-A1 signaling pathway reduces inflammatory factor expression via p38/SR-A1 and decreases MMP9 activity, alleviating CIPN symptoms. Metformin, an AMPK activator, has been shown to lessen mechanical allodynia in mice, underscoring the pathway’s relevance for CIPN treatment (Yang et al., 2023).

Supporting these findings, another study indicates that MMP9 is activated via the HMGB-1/TLR4/PI3K/AKT signaling pathway in macrophages and neurons within the DRG. MMP9 is both a byproduct of neuroinflammation and a contributor to CIPN’s initial phase. The study also endorses the therapeutic utility of NAC in reducing MMP9 activity and neuropathic pain in an oxaliplatin-induced mouse model, with dose-dependent efficacy. NAC’s inhibition of MMP9 reduces calcitonin gene-related peptide expression and suppresses microglial activation in the spinal cords of treated mice, indicating its extensive role in modulating neuroinflammation and central sensitization (Gu et al., 2020).

Research has shown that cisplatin treatment induces peripheral neuropathy, associated with the upregulation of MMP-9 in DRG neurons. This upregulation appears as part of a senescence-like response, where accumulated senescent-like cells in the DRG may contribute to neuropathy. Clearing these senescent cells in animal models has proven effective in reversing CIPN. Although the exact mechanism of MMP-9’s involvement remains to be fully clarified, this finding aligns with observations of MMP-9’s upregulation in other chemotherapy models, like those with oxaliplatin (Acklin et al., 2020).

The evidence from studies examining neuropathic pain and CIPN allows for the speculation that similar mechanisms involving MMP9 may underlie various neuropathic conditions, including CIPN. The observed upregulation of MMP9 following chemotherapy with agents such as paclitaxel and oxaliplatin may contribute to the exacerbation of neuropathic pain in patients with CIPN. This pattern of upregulation aligns with the mechanisms observed in broader neuropathic pain research, indicating that similar pathological processes may be at work in CIPN (Tonello et al., 2019). Nonetheless, the specific triggers associated with chemotherapy may modulate MMP9 activity in ways not seen in other forms of neuropathic pain. While insights from different neuropathic pain studies provide a valuable framework for understanding CIPN, the unique aspects of chemotherapy-induced neuropathy need targeted research. Therefore, additional research is needed to confirm these mechanisms in the context of chemotherapy and to understand any distinct dynamics that may exist.

Nevertheless, building on the understanding that MMP9 plays a role in various forms of neuropathic pain, we can turn our focus mechanisms, which might be significant drivers of CIPN. One critical pathway involves the degradation of myelin basic protein by MMP9, leading to demyelination. This process is significant in the development of neuropathic pain as it exposes axonal membranes and contributes to abnormal pain perception (Chattopadhyay et al., 2007; Kobayashi et al., 2008). In CIPN, the chemotherapy-induced upregulation of MMP9 might similarly initiate or exacerbate demyelination, establishing a direct link between MMP9 activity and the sensory disturbances characteristic of chemotherapy-induced neuropathy (Tonello et al., 2019).

Furthermore, MMP9’s role in modulating the inflammatory response is another indicator that MMP9 could be a driver of neuronal damage across different conditions. MMP9 facilitates the conversion of pro-inflammatory cytokines into their active forms, amplifying the inflammatory environment and contributing to neuronal hyperexcitability. This mechanism, observed in neuropathic pain models, likely contributes to the development and persistence of pain in CIPN as well, where inflammation plays a crucial role in nerve injury (Li et al., 2016; Pan et al., 2018). Additionally, MMP9’s involvement in activating central glial cells, such as astrocytes, through pathways like JNK-MMP9, provides insight into the maintenance of neuropathic pain states (Jiang et al., 2017). This astrocyte activation, contributing to the chronicity of pain, suggests that chemotherapy-induced MMP9 upregulation could similarly extend pain signals in CIPN, reinforcing the broad impact of MMP9 on both peripheral and central aspects of neuropathy.

It becomes evident that despite the relatively narrow scope of studies specifically targeting MMP9 within this context, the findings thus far provide significant insights into its multifaceted role. The introduction of chemotherapeutic agents is associated with increased MMP9 activity, which contributes to neuroinflammation and the degradation of nerve tissue integrity. This series of events demonstrates the critical role of MMP9 in the pathophysiology of CIPN. Interventions aimed at inhibiting MMP9 activity have shown potential in preventing or reversing CIPN symptoms, indicating a valuable approach for reducing chemotherapy’s neurotoxic effects (Tonello et al., 2019). Furthermore, MMP9’s contribution to neuroinflammation and nerve damage through thrombosis-induced circulatory disturbances (Y. Yang et al., 2019), and its regulation by HMGB1 protein, further emphasize its pivotal role in both the onset and progression of CIPN (Gu et al., 2020; Yang et al., 2023).

The intricate involvement of MMP9 within the mechanisms of both general neuropathic pain and CIPN, influenced by direct and indirect regulatory means, makes the enzyme a key target for therapeutic intervention (Gu et al., 2020; Tonello et al., 2019). By aiming to inhibit MMP9’s activity or modulating its upstream activators, it may be possible to provide relief for patients suffering from neuropathic pain or to lessen the neurotoxic side effects associated with chemotherapy (Tonello et al., 2019; Yang et al., 2019). The extensive network of factors that affect MMP9’s activity offers numerous targets for drug development, offering hope for more effective treatments for these debilitating conditions (Gu et al., 2020; Yang et al., 2023).

## 4 MMP9: Shared pathways and mechanisms of neuropathy and colon cancer

MMP9 plays a critical role in both inflammation and tissue remodeling, processes fundamental to the development of colorectal cancer and neuronal damage. This enzyme, known for its ability to break down extracellular matrix components, aids cancer progression by altering the tumor microenvironment (Florin Georgescu et al., 2015) and contributes to neuropathic conditions through changes in neural tissue architecture (Chattopadhyay et al., 2007; Kobayashi et al., 2008).

The link between MMP9 and inflammation is evident from its correlation with inflammatory mediators like IL-6 and CRP, indicating an inflammatory environment that may boost MMP9 levels, thereby facilitating cancer’s advancement (Rasic et al., 2018). Similarly, MMP9’s action on the neuron ECM, triggered by inflammatory stimuli, can disturb the integrity of neural tissues, potentially intensifying neuropathic pain (Kobayashi et al., 2008). MMP9’s regulation by inflammatory cytokines such as IL-1β, TNF-α, and IL-6 is particularly critical in neuropathic pain, as it is associated with central sensitization (Pan et al., 2018).

In tissue remodeling, MMP9’s impact is extensive. In colon cancer, it facilitates tumor cell invasion by breaking down ECM components (Florin Georgescu et al., 2015; Mudatsir et al., 2023). This enzymatic activity extends to the nervous system, influencing myelin basic protein turnover and, consequently, neural structure and function. MMP9- driven myelin degradation can alter nerve transmission and lead to nerve hyperexcitability (Chattopadhyay et al., 2007; Kobayashi et al., 2008). The observed protection against neuropathic pain and preservation of myelin in the absence of MMP9 highlights its significant influence on nerve structure and function (Kobayashi et al., 2008). This sets a foundation for the need of more detailed research into how MMP9 activity shapes neurological health and disease.

Furthermore, signal transduction pathways like PI3K/AKT play a significant role in MMP9 regulation in CRC, influencing tumor cell proliferation, survival, and invasion (Idiiatullina et al., 2021). This regulation indicates a connection between intracellular signaling and the remodeling of the extracellular matrix. The JNK and p38 MAPK pathways, which regulate MMP9-related cellular activities in CRC, are also activated in response to nerve injury in neuropathic pain, leading to increased MMP9 expression and contributing to the inflammatory response and heightened pain (Jiang et al., 2017; Li et al., 2016). Additionally, MMP9’s modulation by the PI3K/AKT pathway in macrophages within the dorsal root ganglia links it to neuroinflammatory responses (Gu et al., 2020), highlighting MMP9’s broad influence in both CRC progression and neuropathic pain pathology.

Macrophages are integral to MMP9-associated pathways in both colorectal cancer and neuropathic pain. They produce MMP9 within the CRC tumor microenvironment, promoting tumor progression and invasion (Florin Georgescu et al., 2015). Similarly, in CIPN, macrophage-induced thrombosis following chemotherapy activates MMP9, pointing to their involvement in neuropathic damage (Yang et al., 2019). Additionally, macrophages’ role in HMGB1 clearance through AMPK activation can indirectly regulate MMP9 expression and activity, influencing neuropathic pain’s inflammatory response (Yang et al., 2023). Thus, macrophages serve as an important intermediary between inflammation, tissue remodeling, and MMP9 activity, contributing to both CRC and neuropathic pain pathology.

## 5 MMP9: A risk factor of chronic CIPN?

**FIGURE 3:**
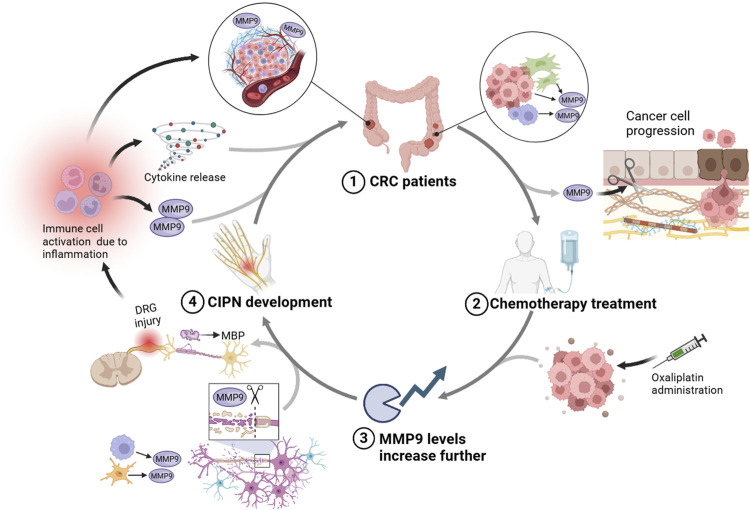
This diagram illustrates a proposed interplay between CRC, CIPN, and the role of MMP9. Starting with (1) CRC patients, the tumor environment inherently produces higher levels of MMP9, contributing to cancer progression. The subsequent (2) chemotherapy treatment, especially with agents like oxaliplatin, is shown to further boost MMP9 levels, which could worsen the ECM degradation and promote tumor spread. (3) This elevated MMP9 activity can contribute to demyelination through the breakdown of MBP, aggravating the sensory dysfunctions and pain associated with CIPN, as indicated by neural injury in the DRG. Consequently, (4) the development of CIPN is marked by DRG damage and myelin sheath breakdown. This neuropathy could trigger further immune cell activation and inflammation, perpetuating a cycle that increases MMP9 expression, thereby connecting back to the initial impact on CRC patients. Created with BioRender.

Concluding on the role of MMP9 as a potential risk factor in the context of CRC and CIPN, the evidence suggests a complex interplay where MMP9 serves as a critical mediator across both conditions. On one hand, elevated MMP9 levels in CRC patients, due to the intrinsic nature of the tumor environment, may predispose these individuals to an increased risk of developing CIPN when subjected to chemotherapy. Chemotherapy’s propensity to elevate MMP9 levels further exacerbates its activity, leading to intensified damage within peripheral nerve tissues. This heightened MMP9 presence, driven by chemotherapy, not only poses a risk for the development of CIPN but also risks exacerbating cancer outcomes by facilitating tumor invasion and metastasis through its remodeling capabilities (Florin Georgescu et al., 2015; Mudatsir et al., 2023). Specifically, the dual action of MMP9 in promoting CRC progression through ECM remodeling and influencing neuropathic pain by altering myelin dynamics presents a complex scenario (Chattopadhyay et al., 2007; Kobayashi et al., 2008). Treatments targeting cancer may inadvertently enhance MMP9 activity, which, while aiming to suppress tumor growth, might also facilitate tumor spread and contribute to the deterioration of nerve health through mechanisms converging on MMP9’s multifunctionality (see Figure 3).

Moreover, the inflammatory response mediated by MMP9 could create a feedback loop. For example, the activation of immune cells leads to the further release of pro-inflammatory cytokines, which in turn can stimulate more MMP9 production. Therefore, the systemic inflammation often seen in CRC patients could be further amplified by chemotherapy treatment, potentially leading to higher levels of MMP9 (Rasic et al., 2018). This dual role of MMP9 in promoting inflammation suggests a vicious cycle where its elevated activity could worsen cancer outcomes and increase the vulnerability of patients to CIPN. As illustrated in Figure 3, this model emphasizes the complex interaction between CRC and CIPN, where MMP9’s multifaceted role perpetuates both cancer progression and neuropathic damage, underlining the need for integrated therapeutic strategies targeting this possible vicious circle. Furthermore, the involvement of MMP9 in modulating macrophage activity within CRC sheds light on a possible mechanism exacerbating CIPN. The MMP9-driven macrophage activity not only supports cancer progression but also sets a precedent for systemic inflammatory responses that could amplify the susceptibility to neuropathic conditions (Gu et al., 2020; Yang et al., 2023).

Lastly, chemotherapy itself, while aiming to eradicate cancer cells, may trigger MMP9 expression, linking cancer treatment directly to the aggravation of neuropathic conditions. Identifying MMP9 levels could help pinpoint patients at increased risk of CIPN, highlighting the importance of treatment strategies that account for MMP9’s dual impact on cancer and neuropathy (Tonello et al., 2019; Yang et al., 2019). Addressing this interplay may improve patient outcomes by simultaneously managing tumor progression and reducing neuropathic complications.

## 6 Conclusions and summary

In summary, this review has explored the relationship between colorectal cancer (CRC) and neuropathy, with a specific focus on chemotherapy-induced peripheral neuropathy (CIPN), highlighting the role of matrix metalloproteinase 9 (MMP9). The findings suggest that MMP9 is involved in both the progression of CRC and the development of neuropathic pain, making it a key factor in understanding the impact of cancer treatment.

MMP9’s role in breaking down the extracellular matrix and promoting tumor growth and spread in CRC has been well documented. Additionally, chemotherapy treatments, especially oxaliplatin, may boost MMP9 levels, potentially worsening neuropathy symptoms in patients (Yang et al., 2023). The connection between MMP9’s involvement in cancer progression and neuropathy points to the critical role the enzyme plays across the spectrum of cancer treatment.

Ultimately, the evidence supports MMP9 as a critical mediator in CRC, aiding tumor invasion and metastasis, and its increased activity due to chemotherapy is suggested as a potential risk for developing CIPN.

The overwhelming majority of (preclinical) studies on CIPN do not consider the effect of cancer growth on neuronal functions. However, there is growing evidence on the robust systemic effects of colon cancer on neurons. As we have shown recently on orthotopic colon cancer mouse models of different genetic backgrounds, the development of the tumors leads to systemic neuronal changes (Balogh et al., 2022). These changes are in great overlap with changes observed in CIPN (e.g., mitochondrial damage or intracellular Ca^2+^ changes in neurons). Vast literature indicates that the increased levels of MMP9 in colon cancer (chapter 2 of this review) might be a driving factor in the development of CIPN (chapter 3). However, until today, there has not been a single study that directly investigates MMP9 (or other plausible risk factors) in both conditions together. This points to a clear gap in the research and indicates that the links suggested between MMP9, colorectal cancer, and neuropathy are, at this stage, hypotheses grounded in existing but separate pieces of evidence.

## 7 Conclusion

The evidence suggests that MMP9 could act as a bridge between colorectal cancer and chronic CIPN development. Further future research could be crucial for understanding the broader implication of cancer treatment, especially the role of chemotherapy in influencing MMP9 activity and, consequently, neuropathic pain outcomes. In the future, screening for elevated serum MMP9 levels in CRC patients might serve as a predictive measure for the risk of CIPN.

All this also indicates the importance of the consideration of cancer-induced alterations in CIPN research. The colon cancer-driven robust systemic inflammatory changes (involving the upregulation of MMP9) seem to induce systemic neuronal alterations and damage, which in turn have major implications regarding the development of CIPN (Balogh et al., 2022).

## Glossary

CRC: colorectal cancer
CIPN: chemotherapy-induced peripheral neuropathy
MMP2/3/9: matrix metalloproteinase 2, 3, and 9
CCL2: chemokine (C-C motif) ligand 2
CXCL1/2/10: chemokine (C-X-C motif) ligand 1, 2 and 10
TIMP: tissue inhibitors of metalloproteinases
ECM: extracellular matrix
IL-6/1β: interleukin-6 and interleukin-1 beta
CRP: C-reactive protein
gp130: glycoprotein 130
JAK2: Janus kinase 2
STAT3: signal transducer and activator of transcription 3
AG490: specific inhibitor of JAK2
USP39: ubiquitin-specific protease 39
TCF4: transcription factor 4
Wnt/β-catenin: Wingless/Integrated and beta-catenin
ERK1/2: extracellular signal-regulated kinase 1 and 2
miR-203: microRNA-203
ETS1: E26 transformation-specific sequence 1
PI3K: phosphoinositide 3-kinase
AKT: protein kinase B
KiSS-1: kisspeptin-1
NF-κB: nuclear factor kappa-light-chain-enhancer of activated B cells
BITC: benzyl isothiocyanate
PKCδ/γ: protein kinase C delta and gamma
JNK1/2: c-Jun N-terminal kinase 1 and 2
MAPK: mitogen-activated protein kinase
AP-1: activator protein 1
Sp-1: specificity protein 1
CA: cholic acid
NADPH: nicotinamide adenine dinucleotide phosphate
ROS: reactive oxygen species
PAR2: protease-activated receptor 2
NAC: N-acetylcysteine
PC: procyanidins
TNF-α: tumor necrosis factor alpha
CCI: chronic constrictive injury
DRG: dorsal root ganglion
NR1: NMDA receptor 1
LPS: lipopolysaccharides
NMDA: N-Methyl-D-aspartate
TAK1: transforming growth factor-beta-activated kinase 1
MBP: myelin basic protein
GM6001: broad-spectrum matrix metalloproteinase inhibitor
CX3CL1: chemokine (C-X3-C motif) ligand 1
CX3CR1/2: chemokine (C-X3-C motif) receptor 1 and 2
CCR2: chemokine (C-C motif) receptor 2
TLR-4: Toll-like receptor 4
SOCS3: suppressor of cytokine signaling 3
HIF-1α: hypoxia-inducible factor 1-alpha
HSP70: heat shock protein 70
HMGB1: high-mobility group box 1
AMPK: AMP-activated protein kinase
SR-A1: scavenger receptor class A member 1
